# Neurons with Cat’s Eyes: A Synthetic Strain of α-Synuclein Fibrils Seeding Neuronal Intranuclear Inclusions

**DOI:** 10.3390/biom12030436

**Published:** 2022-03-11

**Authors:** Francesca De Giorgi, Muhammed Bilal Abdul-Shukkoor, Marianna Kashyrina, Leslie-Ann Largitte, Francesco De Nuccio, Brice Kauffmann, Alons Lends, Florent Laferrière, Sébastien Bonhommeau, Dario Domenico Lofrumento, Luc Bousset, Erwan Bezard, Thierry Buffeteau, Antoine Loquet, François Ichas

**Affiliations:** 1 Institut des Maladies Neurodégénératives, CNRS, UMR 5293, 33076 Bordeaux, France; leslie-ann.largitte@u-bordeaux.fr (L.-A.L.); florent.laferriere@u-bordeaux.fr (F.L.); erwan.bezard@u-bordeaux.fr (E.B.); 2 Institut des Maladies Neurodégénératives, UMR 5293, Université de Bordeaux, 33076 Bordeaux, France; 3 Institut de Chimie et de Biologie des Membranes et des Nano-objets, CNRS, UMR 5248, Université de Bordeaux, 33600 Pessac, France; mb.abdul-shukkoor@iecb.u-bordeaux.fr (M.B.A.-S.); a.lends@iecb.u-bordeaux.fr (A.L.); a.loquet@iecb.u-bordeaux.fr (A.L.); 4 Institut Européen de Chimie et Biologie, Université de Bordeaux, 33600 Pessac, France; 5 Department of Biological and Environmental Sciences and Technologies, Section of Human Anatomy, University of Salento, 73100 Lecce, Italy; marianna.kashyrina@unisalento.it (M.K.); francesco.denuccio@unisalento.it (F.D.N.); dario.lofrumento@unisalento.it (D.D.L.); 6 Institut Européen de Chimie et Biologie, CNRS, Université de Bordeaux, INSERM, UMS3033/US001, 33600 Pessac, France; b.kauffmann@iecb.u-bordeaux.fr; 7 Institut des Sciences Moléculaires, CNRS, UMR 5255, Université de Bordeaux, 33400 Talence, France; sebastien.bonhommeau@u-bordeaux.fr (S.B.); thierry.buffeteau@u-bordeaux.fr (T.B.); 8 Laboratory of Neurodegenerative Diseases, Institut François Jacob, MIRCen, CEA, CNRS, 92265 Fontenay-aux-Roses, France; luc.bousset@cnrs.fr

**Keywords:** α-Synuclein, amyloid, fibril, strain, neuron, nuclear, inclusion, lentiform, cat eye, Multiple System Atrophy, Parkinson’s disease

## Abstract

The distinct neuropathological features of the different α-Synucleinopathies, as well as the diversity of the α-Synuclein (α-Syn) intracellular inclusion bodies observed in post mortem brain sections, are thought to reflect the strain diversity characterizing invasive α-Syn amyloids. However, this “one strain, one disease” view is still hypothetical, and to date, a possible disease-specific contribution of non-amyloid factors has not been ruled out. In Multiple System Atrophy (MSA), the buildup of α-Syn inclusions in oligodendrocytes seems to result from the terminal storage of α-Syn amyloid aggregates first pre-assembled in neurons. This assembly occurs at the level of neuronal cytoplasmic inclusions, and even earlier, within neuronal intranuclear inclusions (NIIs). Intriguingly, α-Syn NIIs are never observed in α-Synucleinopathies other than MSA, suggesting that these inclusions originate (i) from the unique molecular properties of the α-Syn fibril strains encountered in this disease, or alternatively, (ii) from other factors specifically dysregulated in MSA and driving the intranuclear fibrillization of α-Syn. We report the isolation and structural characterization of a synthetic human α-Syn fibril strain uniquely capable of seeding α-Syn fibrillization inside the nuclear compartment. In primary mouse cortical neurons, this strain provokes the buildup of NIIs with a remarkable morphology reminiscent of cat’s eye marbles (see video abstract). These α-Syn inclusions form giant patterns made of one, two, or three lentiform beams that span the whole intranuclear volume, pushing apart the chromatin. The input fibrils are no longer detectable inside the NIIs, where they become dominated by the aggregation of endogenous α-Syn. In addition to its phosphorylation at S129, α-Syn forming the NIIs acquires an epitope antibody reactivity profile that indicates its organization into fibrils, and is associated with the classical markers of α-Syn pathology p62 and ubiquitin. NIIs are also observed in vivo after intracerebral injection of the fibril strain in mice. Our data thus show that the ability to seed NIIs is a strain property that is integrally encoded in the fibril supramolecular architecture. Upstream alterations of cellular mechanisms are not required. In contrast to the lentiform TDP-43 NIIs, which are observed in certain frontotemporal dementias and which are conditional upon GRN or VCP mutations, our data support the hypothesis that the presence of α-Syn NIIs in MSA is instead purely amyloid-strain-dependent.

## 1. Introduction

Parkinson’s disease (PD), Multiple System Atrophy (MSA), and Dementia with Lewy Bodies (DLB) are the three key clinical entities constituting α-Synucleinopathies [1]. They represent a class of neurodegenerative diseases characterized by the emergence and the multiplication of intracellular α-Syn amyloid inclusions in neuronal and non-neuronal cells [2]. These inclusions appear in the wake of the neurodegenerative process, and whether they cause or instead simply beacon the cell’s demise is still debated [3,4]. Supporting a causal role, however, is the striking resemblance of α-Syn amyloids with prions [5,6,7]: beyond peptide sequence homologies of their fibrillization-prone domain [5], α-Syn amyloids can seed their own templated growth [5,8,9,10,11,12,13,14], resulting in a high-fidelity replication of pathological α-Syn amyloid strains in neurons [15,16]. This is in line with the reference neuropathology studies which evidenced a gradual invasion of anatomically connected brain structures during disease progression, here also suggesting that α-Synucleinopathies obey a prion-like etiologic mechanism [17,18,19,20].

According to this view, (i) α-Syn amyloid aggregation accidentally originates in a few susceptible neurons [6,21,22], (ii) second to buildup, α-Syn fibril fragments are released or leak out [20], (iii) healthy bystander neurons take up the latter fragments which (iv) seed a new cycle of templated amyloid aggregation [5,6] that eventually kills the cell, causing a new release of fragments, and so on [20]. Different α-Syn fibril strains [15,23,24,25,26,27,28] could affect the characteristics of every single step [15,29,30]. Thus, just as different prion strains cause different human disease phenotypes [31], distinct α-Syn fibril strains could be at the origin of PD, DLB and MSA [32].

This hypothesis is substantiated by the presence of disease-specific α-Syn fibril morphologies and assemblies in brain extracts [27,33,34]. Therefore, the anatomical origin of the spatial spread, its dynamics, and the affected brain regions have all attracted enormous interest to experimentally identify links between disease progression features and specific α-Syn fibril strains [15,35,36,37,38,39,40]. For recent reviews see [41,42].

In MSA, a growing body of evidence [43,44,45,46,47] indicates that the numerous α-Syn inclusions found in oligodendrocytes [48,49] represent a late by-product of a fibrillization process taking place in neurons [43,44,45,48], at the term of which assembled aggregates are handed over to oligodendrocytes for inert storage [43]. In other words, early phases of MSA involve a neuronal α-Synucleinopathy [44,45,47].

We recently isolated a new synthetic strain of human α-Syn fibrils [15] characterized by (i) an extremely poor staining with Thioflavin T (ThT), (ii) a propensity to induce a massive neuronal α-Syn pathology in vivo, and most strikingly, (iii) a specific ability to seed phosphorylated α-Syn aggregates crisscrossing neuronal nuclei [15], i.e., α-Syn neuronal intranuclear inclusions (NIIs) [50]. These three characteristics point to MSA [44,45,50,51], with NIIs even constituting a specific hallmark of this disease [50]. Thus, NIIs could reveal strain-encoded properties of the α-Syn fibrils specifically found in MSA [27,34]. Because of this analogy, we undertook a thorough biophysical characterization of our new synthetic strain and proceeded with a detailed cytological analysis of the experimental NIIs in vitro and in vivo.

## 2. Materials and Methods

### 2.1. α-Syn Expression

Escherichia coli strain BL21(DE3) plysS was transformed with pET24–α-Syn vector by electroporation and plated onto a Luria broth agar plate containing kanamycin (30 μg/mL). A preculture in 5 mL of Luria broth medium was inoculated with one clone and incubated at 37 °C under 200 rpm shaking for 4 h. The expression on α-Syn was carried out in M9 minimal medium containing ^13^C glucose (2 g/L) and ^15^NH4Cl (1 g/L) as carbon and nitrogen sources. Cells from the Luria broth preculture were recovered by centrifugation (1000× *g*, 10 min) and used for inoculating 200 mL of M9 medium. Cells were grown overnight at 37 °C under 200 rpm shaking and then diluted in 2 L of culture. Protein expression was induced by adding 1 mM isopropyl-β-d-thiogalactopyranoside during the exponential phase, evaluated at an optical density at 600 nm reaching 0.8. Cells were harvested after 4 to 5 h of culture at 37 °C by 6000× *g* centrifugation (JLA 8.1000, Beckman Coulter, Villepinte, France), and pellet was kept at −20 °C before purification.

### 2.2. α-Syn Purification

The pellets were resuspended in lysis buffer (10 mM Tris and 1 mM EDTA (pH 7.2) with protease inhibitor tablets (cOmplete, EDTA-free protease inhibitor cocktail, Roche, Basel, Swiss)) and sonicated at 50% max energy, 30 s on and 30 s off for three rounds with a probe sonicator (Q-Sonica, Newtown, CT, USA). The sonicated pellets were centrifuged at 20,000× *g* for 30 min, and the supernatant was saved. The pH of the supernatant was then reduced to pH 3.5 using HCl, and the mixture stirred at room temperature (RT) for 20 min and then centrifuged at 60,000× *g* for 30 min. The pellets were discarded. The pH of the supernatant was then increased to pH 7.4 with NaOH and then dialyzed against 20 mM tris-HCl (pH 7.40) and 100 mM NaCl buffer before loading onto a 75 pg HiLoad 26/600 Superdex column equilibrated with the same buffer with ÄKTA pure system. Monomeric fractions were collected and concentrated if needed by using Vivaspin 15R 2-kDa cutoff concentrator (Sartorius Stedim, Göttingen, Germany). Purification fractions were checked by using polyacrylamide gel electrophoresis (PAGE) tris-tricine 13% dying with ProBlue Safe Stain. Protein concentration was evaluated spectrophotometrically by using absorbance at 280 nm and extinction coefficient of 5960 M^−1^ cm^−1^.

### 2.3. α-Syn Fibrillization

Solutions of monomeric α-Syn at 4 to 5 mg/mL in saline (H_2_O, 100 mM NaCl, and 20 mM tris-HCl (pH 7.40)) were sterilized by filtration through 0.22 μm Millipore single-use filters and stored in sterile 15 mL conical falcon tubes at 4 °C. Sterilized stock was then distributed into safe-lock Biopur individually sterile-packaged 1.5 mL Eppendorf tubes as 500 μL aliquots. For the present work, 6 tubes were seeded with 1% of the α-Syn 1B fibrils described in [15]. The tubes were cap-locked and additionally sealed with parafilm. All previous steps were performed aseptically in a particle-free environment under a microbiological safety laminar flow hood. All samples were loaded simultaneously in a ThermoMixer (Eppendorf) in a 24-position 1.5 mL Eppendorf tube holder equipped with a heating lid. Temperature was set to 37 °C, and continuous shaking at 2000 rpm proceeded for 4 days.

### 2.4. Sonication

When relevant, 100 μL 1B or “Type 2” (iso1 assemblies from [15]) α-Syn fibril stocks (4 mg/mL) or dilutions to 0.1 mg/mL in 100 μL in PBS were distributed in cap-locked, sterile 0.5 mL polymerase chain reaction (PCR) tubes (Thermo Fisher Scientific, Bordeaux, France). Sonication was performed at 25 °C in a Bioruptor Plus water bath sonicator (Diagenode, Liège, Belgium) equipped with thermostatic control and automated tube carousel rotator. The sonication power was set to “high,” and 10 cycles of 30 s on followed by 10 s off were applied.

### 2.5. Electron Microscopy

Samples from the fibrillized α-Syn aliquots were diluted to 0.1 mg/mL in MilliQ water. Glow discharged carbon coated 400 mesh copper grids were floated over 30 µL drop for 1 min. The excess of liquid was absorbed on Whatman filter paper, and the grid was stained for 1 min over a 30 µL droplet of 1% uranyl acetate or 2% ammonium molybdate. The grids were observed under Jeol 1400 electron microscope at 80 kV and 10 K magnification, images were recorded on a Gatan Rios CCD camera with automatic drift correction.

### 2.6. X-ray Diffraction

The amyloid diffraction pattern was measured at 290 K on a Rigaku FRX rotating anode X-ray generator at the copper wavelength (Kα, λ = 1.54 Å). The source is equipped with Osmic Varimax HF optics and a Dectris© Eiger 1M detector on a 2θ arm of a Rigaku partial chi AFC11 goniometer. The sample was mounted in a MicroLoop from MiTeGen on a goniometer head under the cryostream nitrogen flux. The diffraction pattern corresponds to a 360° rotation along the phi axis (perpendicular to the direct beam with omega and chi axes at the 0 position) with an exposure time of 360 sec. Data were integrated with CrysalisPro (Rigaku Oxford Diffraction, Ltd., Yarnton, Oxfordshire, UK) with median filter and baseline correction.

### 2.7. Magic-Angle Spinning NMR

NMR experiment was performed at a magic-angle spinning (MAS) frequency of 11 kHz on a triple resonance 3.2 mm MAS probe (Bruker Biospin, Rheinstetten, Germany) using 600 MHz ^1^H Larmor frequency spectrometer (Bruker Biospin, Rheinstetten, Germany). Chemical shifts were calibrated using DSS as an internal reference. The rigid part of the fibrils was probed using two-dimensional ^13^C-^13^C correlation spectrum with a 1 ms long cross-polarization time and a PDSD mixing time of 50 ms. The spectrum was acquired with t_1_ = 9.1 ms, t_2_ = 18.4 ms, recycle delay of 2.5 s and 128 number of scans, leading to a total experimental time of ~1.5 days. The 90 kHz SPINAL-64 decoupling was applied for both dimensions. The spectrum was processed using Bruker Topspin 3.6.1 QSine SSB = 3 apodization functions for both dimensions and analyzed using the CcpNmr 2.4.2 program [52].

### 2.8. Fourier-Transform Infra-Red (FTIR) Spectroscopy

In order to avoid the intense absorption peaks of H_2_O in the 1500–1800 cm^−1^ and the 3000–4000 cm^−1^ regions, saline D_2_O buffer was used as a solvent for all Infra-Red (IR) and Vibrational Circular Dichroism (VCD) measurements. IR spectra of (i) monomeric α-Syn, and monomeric α-Syn seeded with either (ii) 1% of the common “Type 2” human α-Syn fibrils (iso1 assemblies from [15]) or (iii) with 1% 1B α-Syn fibrils were recorded with a ThermoNicolet Nexus 670 FTIR spectrometer (ThermoNicolet, Madison, WI, USA) at a resolution of 4 cm^−1^, by coadding 50 scans. Samples (volume ~35 μL) were held in a demountable CaF_2_ cell with fixed path length of 55 μm (BioTools, Jupiter, FL, USA). IR spectra were performed for three concentrations of monomeric α-Syn (5, 7 and 9 mg/mL) at 25 °C. For each sample, the measurement of IR spectra was repeated at different times for 5 days. All IR spectra have the D_2_O solvent contribution subtracted out, and the baseline corrected. Additional VCD measurements of monomeric α-Syn were performed using a ThermoNicolet Nexus 670 FTIR spectrometer equipped with a VCD optical bench [53]. In this optical bench, the light beam was focused on the sample by a BaF_2_ lens (191 mm focal length), passing an optical filter, a BaF_2_ wire grid polarizer (Specac, Orpington, UK), and a ZnSe photoelastic modulator (Hinds Instruments, Hillsboro, OR, USA, Type II/ZS50). The light was then focused by a ZnSe lens (38.1 mm focal length) onto a 1 × 1 mm^2^ HgCdTe (ThermoNicolet, MCTA* E6032) detector. VCD spectra were recorded at a resolution of 4 cm^−1^, by coadding 24,000 scans (8 h acquisition time). Baseline corrections of the VCD spectra were performed by subtracting the raw VCD spectra of D_2_O solvent. The photoelastic modulator was adjusted for a maximum efficiency in the mid-IR region at 1400 cm^−1^. Calculations were performed via the standard ThermoNicolet software (Omnic™), using Happ and Genzel apodization, de-Haseth phase-correction and a zero-filling factor of 1.

### 2.9. In Vitro α-Syn Pathology, High Content Analysis (HCA) and Laser-Scanning Confocal Microscopy (LSCM)

Timed pregnant C57BL/6J female mice were bred at the animal facility of the IMN. Cortices were harvested from embryonic day 18 mouse embryos and dissociated enzymatically and mechanically (using neural tissue dissociation kit, C Tubes, and an Octo Dissociator with heaters; Miltenyi Biotec, Bergish-Gladbach, Germany) to yield a homogenous cell suspension. The cells were then plated at 20,000 per well in 96-well plates (Corning BioCoat poly-d-lysine imaging plates) in neuronal medium (MACS Neuro Medium, Miltenyi Biotec, Bergish-Gladbach, Germany) containing 0.5% penicillin-streptomycin, 0.5 mM alanyl-glutamine, and 2% NeuroBrew supplement (Miltenyi Biotec, Bergish-Gladbach, Germany). The cultures were maintained with 5% CO_2_ at 37 °C in humidified atmosphere. The medium was changed by one-third every 3 days, until a maximum of 30 DIV (days in vitro). In such cultures neurons represent approximately 85 to 95% of the cell population. After 7 DIV, vehicle or extemporaneously sonicated 1B α-Syn fibrils were added at a final concentration of 10 nM (equivalent monomeric α-Syn concentration). At DIV 30 the plates were aldehyde-fixed, processed for immunofluorescence, and acquired with an IncucyteS3 automated high-content imager as previously described [46]. Multichannel fluorescence optical sections of the samples were performed (thickness < 0.8 µm) using a Leica SP5 LSCM equipped spectral detector, with 488, 561 and 633 nm laser lines, with a motorized X-Y stage and with a mixed stepping motor/piezo Z controller. For 3D reconstructions, volume rendering, in silico sectioning and animations, raw 2-channel Z-stack images were processed offline using icy 2.3 [54] without prior image filtering or noise removal. All immunofluorescence stainings were performed as described in [15,46]. Primary antibodies and dilutions are as follows. Antibody Pair #1 (Figures 3 and 4): Anti-MAP2 antibody Abcam, Cambridge, UK (ab32454) 1:500 and Anti-pS129 phospho-synuclein, Clone pSyn#64, Wako (014-20281), 1:500; Antibody Pair #2 (Figures 4, 8 and 9 and visual abstract movie): mouse monoclonal antibody 4C11 to Lamin A + C (ab238303) 1: 250 and EP1536Y anti-pS129 phospho-synuclein Abcam ab51253 1:500; Antibody Pair #3 (Figure 5A): EP1536Y anti-pS129 phospho-synuclein Abcam ab51253 1:500, and fluorescently coupled MJFR1, anti-human alpha-synuclein (Alexa Fluor® 488) ab195025 1:250; Antibody Pair #4 (Figure 5B): SynF1, anti-aggregated alpha-synuclein BioLegend 847802 1: 500, and D37A6 anti-murine alpha-synuclein, Cell Signaling, #4179 1:500; Antibody Pair #5 (Figure 5C) EP1536Y anti-pS129 phospho-synuclein Abcam ab51253 1:500, and Syn1 clone 42 anti-human and murine alpha-synuclein BD Biosciences 610787 1:500; Antibody Pair #6 (Figure 6A): SynF1, anti-aggregated alpha-synuclein BioLegend 847802 1:500 and Anti-SQSTM1/p62 antibody (2C11) Abcam, (ab56416) 1:500; Antibody Pair #7 (Figure 6B): SynF1, anti-aggregated alpha-synuclein BioLegend 847802 1:500 and anti-ubiquitin PA316717 Fisher Scientific antibody 1:500; Antibody Pair #7 (Figure 6B): SynF1, anti-aggregated alpha-synuclein BioLegend 847802 1:500 and EP1536Y anti-pS129 phospho-synuclein Abcam ab51253 1:500; all other single immunostainings combined with DRAQ7: EP1536Y anti-pS129 phospho-synuclein Abcam ab51253 1:500. DRAQ7 is a fluorescent anthracyclin DNA-intercalating dye and was used according to the manufacturer’s instructions (Thermo Fisher Scientific, Bordeaux, France).

### 2.10. In Vivo α-Syn Pathology and Histology

Adult male C57BL/6J mice were bred at the animal facility of the IMN. All mice were housed in a temperature-controlled (22 °C) and light-controlled environment on a 12 h light/12 h dark cycle with access to food and water ad libitum. The study design was approved by French Ministry of Research and all experimental procedures were conducted in accordance with the European Communities Council Directive (2010/63/EU) for care of laboratory animals. The mice (6–8 weeks old) unilaterally received 2 μL of sonicated α-Syn fibrils 1B (4 mg/mL) by stereotactic delivery at a flow rate of 0.4 μL/min, and the pipette was left in place for 5 min after injection to avoid leakage. Delivery was either (i) in the region immediately above the right substantia nigra (coordinates from bregma: AP, −2.9; L, −1.3; DV, −4.5), n = 10 mice plus 10 sham controls, or (ii) within the right striatum (AP, −0.1; L, +2.5; DV, +3.8), n = 7 mice plus 3 controls. Animals were euthanized after 1 (striatal injection) or 4 months (substantia nigra injections). For the substantia nigra injections, the brains were perfused with saline, postfixed for 3 days in 10 mL of 4% PFA at 4 °C, cryoprotected in gradient 20% sucrose in PBS before being frozen by immersion in a cold isopentane bath (−60 °C) for at least 5 min, and stored immediately at −80 °C until sectioning. After serial cryo-sectioning (50 µm), the sections of interest were stained using the primary antibody EP1536Y (Abcam) for detecting phospho-S129–positive α-Syn amyloid aggregates (dilution 1:5000 for immunohistochemistry (IHC) and 1:500 for immunofluorescence (IF)). The slides were acquired using a Pannoramic slide scanner for IHC, and by LSCM (see higher) for IF. For the striatal injections mice were transcardially perfused with tris-buffered saline (pH = 7.4) followed by 4% paraformaldehyde in PBS pH = 7.4 at 4 °C. Brains were subsequently postfixed in the same fixative, paraffin embedded, and 10 µm sections were obtained with a rotative microtome (Leica, Milan, Italy). IHC was performed following a standard avidin–biotin complex procedure. Briefly, specimens were incubated overnight at 4 °C (dilution 1:2000) with the rabbit monoclonal primary antibody EP1536Y (Abcam) for detecting phospho-S129–positive α-syn amyloid aggregates and then with an anti-rabbit biotinylated secondary antibody (Merck Life Science S.r.l., Milan, Italy), at a 1:1000 dilution for 1 h at room temperature. The antigen–antibody complexes were visualized by incubating the sections for 1 h with extravidin peroxidase (Merck Life Science S.r.l., Milan, Italy) diluted 1:2000 and 3,3′-diaminobenzidine oxidation in the presence of H_2_O_2_. Images were acquired with a Nikon Eclipse E800 microscope equipped with a DS-5M digital camera (Nikon Instruments S.p.A, Campi Bisenzio, FI, Italy).

## 3. Results

### 3.1. Biophysical Characterization of the Synthetic Human α-Syn Fibril Strain 1B

**Figure 1:**
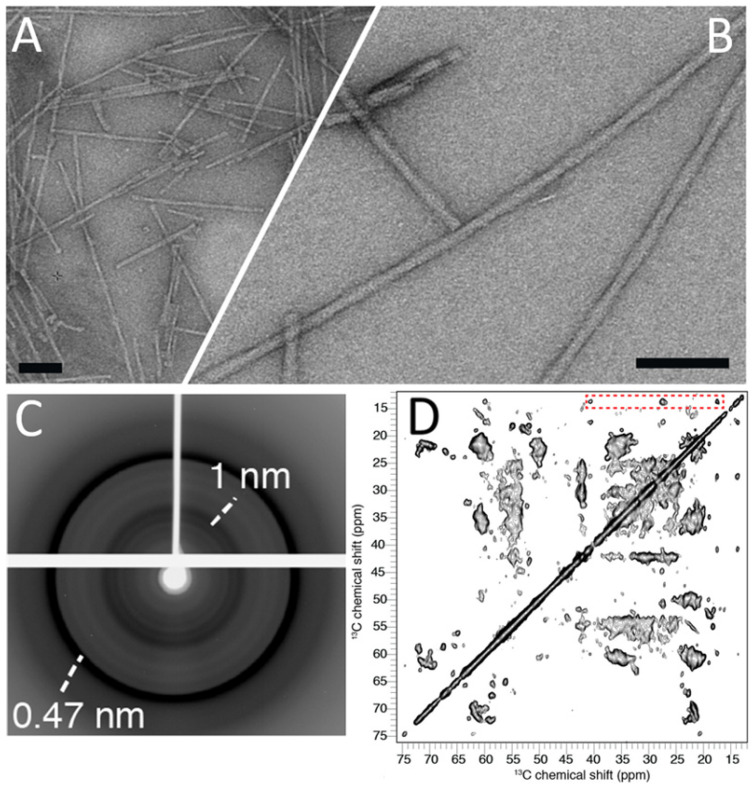
(**A**,**B**) Transmission Electron Microscopy of 1B fibrils at two magnifications (scale bars: 100 nm); (**C**) X-Ray diffraction pattern of unaligned 1B fibrils, reflections at 4.7 and 10 Å are highlighted; (**D**): two-dimensional MAS NMR ^13^C-^13^C correlation experiment probing the residues of the rigid amyloid core of 1B fibrils.

Strain 1B was previously selected by serendipity as a “ThT-negative” human α-Syn fibril strain obtained by the shaking of purified recombinant human α-Syn monomers [15]. The specific characteristics of the 1B strain could be perpetuated in vitro by cascade seeding with 1% of 1B seeds [15]. Thus, we re-generated isotope-labeled 1B fibrils using ^13^C- and ^15^N-labeled human wild-type α-Syn monomers seeded with 1% of unlabeled 1B fibrils in order to obtain a versatile 1B fibril batch amenable to parallel biophysical and biological investigations. From an ultrastructural point of view, the resulting 1B fibrils appeared to be exclusively populated by compact rod-like fibrils composed of two intertwined protofilaments (Figure 1A,B), with a filament crossover periodicity of 98 ± 9 nm (N = 30), a maximal width of 17 ± 1.4 nm (N = 21) and a width at crossover of 13 ± 1 nm (N = 17). This organization is in complete agreement with their X-ray diffraction pattern that indicates a typical cross-β architecture [55] with an interstrand spacing of 0.47 nm and an inter β-sheet distance of 1 nm (Figure 1C). Under cryoEM conditions, 1B fibrils in amorphous ice exhibited a maximal width of 11 nm which is close to the value generally described for classical rod-type synthetic filaments [56].

To further probe the local molecular conformation of 1B, we employed magic-angle spinning (MAS) NMR. We recorded a two-dimensional 50 ms PDSD ^13^C-^13^C correlation experiment to probe the residues of the rigid amyloid core of 1B. The resulting spectrum (Figure 1D) shows the presence of relatively well-resolved ^13^C NMR signals, indicative of a minimal degree of structural polymorphism. For instance, the ^13^C line widths of I88 side chains (dashed box) are ~0.8 ppm, i.e., a width comparable to other α-Syn fibril preparations previously studied by MAS NMR [25,57,58,59]. We compared the ^13^C-^13^C spectrum obtained for 1B with the ^13^C-^13^C spectra of the reference fibril types assembled in vitro using wild-type human α-Syn, and found that 1B differs from ““Type 1” [24,25], “Type 2” [60], and “Neither Type 1 or 2” [61], according to the nomenclature of [62], as well as from “high pH” fibrils [63] (Supplementary Figure S1).

**Figure 2:**
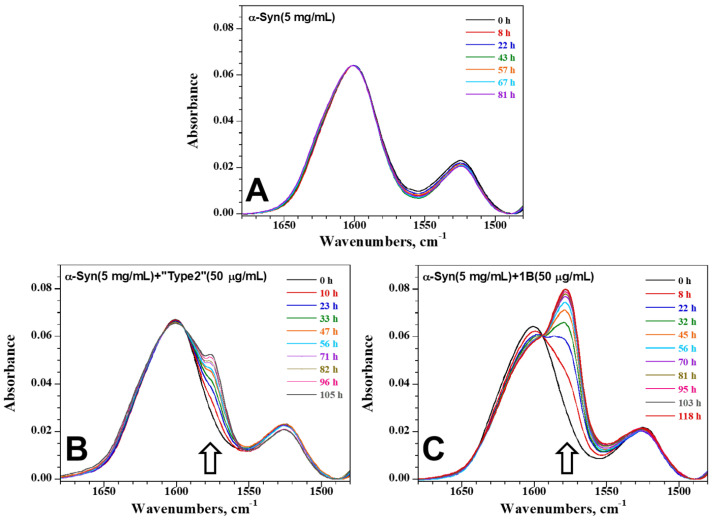
Time lapse of the IR spectra of monomeric α-Syn: (**A**) without seeding, (**B**) seeded with 1% of “Type 2” fibrils, or (**C**) seeded with 1% of 1B fibrils.

In order to see if its distinctive supramolecular architecture could confer functional properties to 1B, we studied the seeding propensity and the fibrillization kinetics of 1B by monitoring it over 5 days in unstirred and air-tight encased thin films of ^13^C ^15^N α-Syn monomer solutions using FTIR spectroscopy [64]. First, we investigated the spontaneous evolution of the IR spectra of monomeric α-Syn in time. Figure 2A and Supplementary Figure S2 show the IR spectra in the amide I’ and amide II’ region (1680–1480 cm^−1^) at different time points for 5 days. The broad amide I’ band, located around 1600 cm^−1^ for monomeric α-Syn labeled with ^13^C and ^15^N, originates mostly from the peptide bond ^13^C=O stretching vibration and is sensitive to the secondary structure of the protein. Due to the ^13^C-labeling of α-Syn, we observe a downshift of the amide I’ band by ca. 45 cm^−1^ with respect to the amide I band related to the ^12^C=O stretching vibration [65]. The spectral modifications due to conformational changes are the same as those appearing for unlabeled protein but they are also downshifted by nearly 45 cm^−1^. Thus, the amide I’ spectral pattern observed in Figure 2A can be interpreted as representative of a disordered and/or random coil protein. The VCD pattern observed in Supplementary Figure S3, with negative and positive peaks at 1590 cm^−1^ and 1620 cm^−1^, respectively, shows that monomeric α-Syn contains 3_1_-helix structures in solution [66], i.e., indicating a random coil structuration. Figure 2A and Supplementary Figure S2 show that in the absence of seeding, no modification of the IR spectra could be observed over 5 days for two concentrations (5 and 9 mg/mL) of monomeric α-Syn, indicating that unseeded monomers did not self-assemble into fibrils. The changes in IR spectra in the amide I’ region for monomeric α-Syn seeded with 1% of the common “Type 2” [62] (Figure 2B, Supplementary Figure S4) and 1B (Figure 2C, Supplementary Figure S5) fibrils are different from that observed for monomeric α-Syn alone, with the appearance of a new component at 1575 cm^−1^ which increases over time (empty arrows). This component is typical of intermolecular β-sheets resulting from self-aggregation of monomers occurring during fibrillization. Comparison of the two seeded conditions indicates that 1B is much more prone to seed and to template α-Syn monomers and/or that the fold of α-Syn into the 1B fibrils presents more extended regions structured as “in register” β-strands compared to “Type 2” fibrils. Whatever the option, these FTIR results: (i) are in agreement with our observation that 1B α-Syn fibrils are capable of seeding a profuse α-Syn pathology in neurons while the “Type 2” fibrils are barely active [15], and (ii) correlate with the differential staining of the two fibril types with ThT (Supplementary Figure S6). The ~10 times higher ThT staining of “Type 2” fibrils parallels their negligible seeding activity in FTIR experiments and in neurons [15] compared to the 1B strain.

### 3.2. Neuronal Inclusions Seeded by 1B α-Syn Fibrils

**Figure 3:**
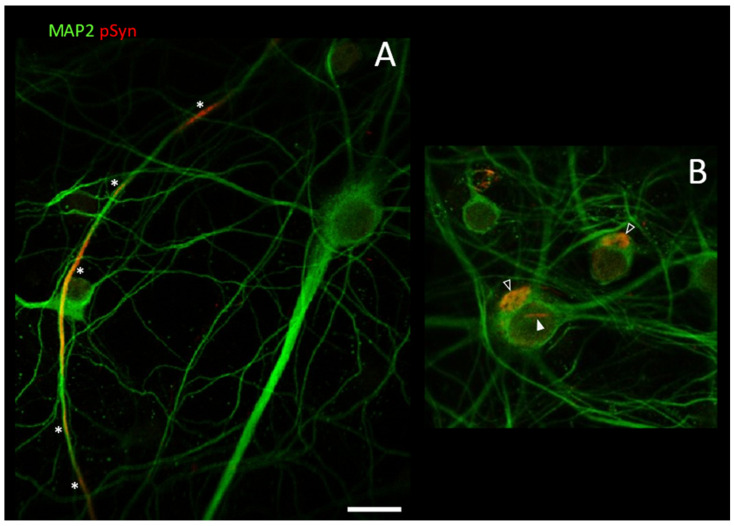
Laser-Scanning Confocal Microscopy (LSCM) optical sections of DIV30 mouse primary cortical neurons treated at DIV7 with 1B fibrils. Phospho-S129 α-Syn-positive inclusions are coded in red (asterisks: neuritic inclusions; empty arrows: NCIs; plain arrow: NII). Neuron-specific cytoplasmic immuno-staining against MAP2 coded in green. Scale bar: 10 µm. For description of (**A**,**B**), see text.

We previously reported [15] that in primary cultures of cortical neurons, the α-Syn pathology seeded by 1B consisted in the formation of numerous neuritic and neuronal cytoplasmic inclusions (NCIs), but also—and most specifically—of NIIs (quantifications in [15] and at the end of the Section 3.3.). Figure 3 shows two confocal sections that document the different types of α-Syn inclusions that can be observed by Laser-Scanning Confocal Microscopy in individual cortical neurons in culture at DIV30, 23 days after treatment with 10 nM of 1B fibrils.

In this figure, the cytoplasm of the neuronal soma and processes is specifically revealed using a primary antibody recognizing the neuronal marker MAP2 (green). The neuronal nuclei are easily identified as negative voids delineated by MAP2. The intraneuronal α-Syn inclusions are revealed using a primary antibody recognizing phospho-S129 α-Syn (red) [8,10,62]. In the confocal section of Figure 3A, two healthy neurons can be seen with many surrounding processes. One of the latter contains several linear phospho-S129 α-Syn-positive segments (white asterisks). These elongated structures are neuritic inclusions. In the confocal section of Figure 3B, instead, two neurons containing somatic inclusions can be seen: the empty arrowheads point to α-Syn NCIs; the white arrowhead indicates a “single-beam” NII in a neuron also containing an NCI. In these primary cultures challenged with 1B, α-Syn nuclear inclusions are not encountered in non-neuronal cells.

### 3.3. NIIs Seeded by 1B

**Figure 4:**
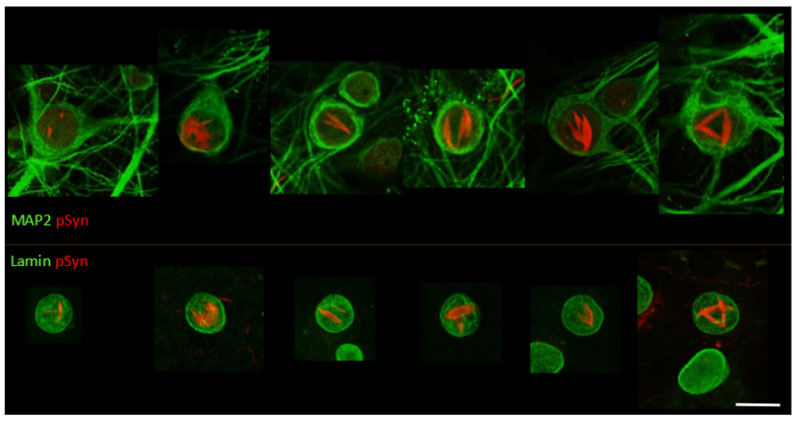
LSCM optical sections of 30 DIV mouse primary cortical neurons treated at DIV7 with 1B fibrils. Phospho-S129 α-Syn-positive inclusions (NIIs) are coded in red; upper row: MAP2 is in green; lower row: Lamin is in green. Scale bar: 10 µm.

We next scanned a series of culture wells treated with 1B to identify the various α-Syn NIIs’ morphologies (Figure 4). In a subset of culture wells, we used MAP2 staining (green) to qualify the neuronal location of the α-Syn NIIs (red) (individual neurons shown in the upper row of Figure 4, six confocal sections), and in other wells we used the nuclear marker Lamin staining (green) to determine the nuclear location of the α-Syn NIIs (red), (individual nuclei shown in the lower row of Figure 4, six confocal sections).

The collection of 12 single-cell confocal sections of Figure 4 shows that α-Syn NIIs can be composed of one but most often of two or three intranuclear lentiform beams that span the whole nucleus, and cross each other forming giant patterns. The “three-beam” NIIs can sometimes be spectacular and organize themselves as planar geometrical structures: triangles or “loose three crescent moons”. Another example is shown in the video abstract (https://doi.org/10.5281/zenodo.6338439) with the animation of a 3D volume rendering of a Lamin (white) and phospho-S129 α-Syn (red) Z-stack of an NII forming a cross that spans the whole nuclear volume. In these images and movie, the neuronal nuclei harboring NIIs are morphologically reminiscent of “cat’s eye” marbles. The neurons harboring NIIs in random samples of the 1B-treated cultures (n = 30) represent 48% of the total number of neurons harboring phospho-S129 α-Syn-positive somatic inclusions (i.e., soma with an NII and/or an NCI) (n = 92). The neurons harboring an NII and/or an NCI represent 8% of the total neuronal population (n = 1331). Corresponding control cultures treated with α-Syn monomers (n = 30) showed no phospho-S129 α-Syn-positive NIIs, NCIs, or neuritic inclusions. As to other synthetic fibril strains, the capability to seed lentiform NIIs was never reported before [8,10,26,28,32,36,62,63,67,68].

### 3.4. NIIs Are Amyloid Assemblies of Endogenous α-Syn

**Figure 5:**
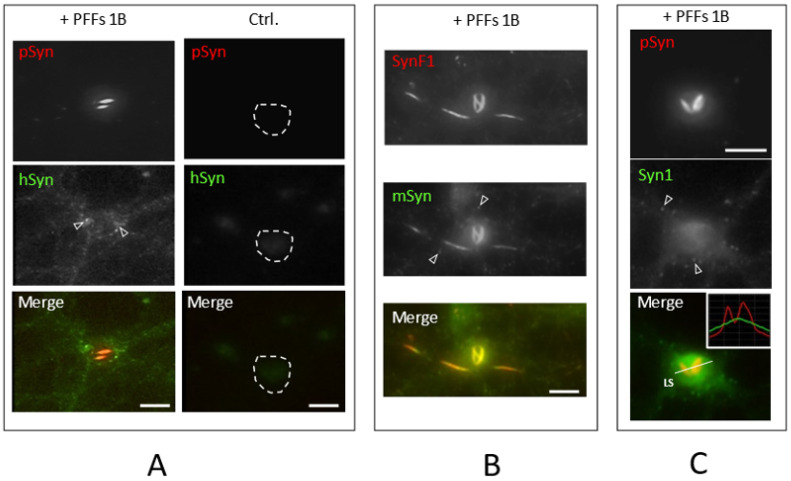
The 20× HCA images of DIV30 mouse primary cortical neurons treated at DIV7 with 1B fibrils (+ PFFs 1B) or solvent only (Ctrl.). pSyn: phospho-S129 α-Syn; human and mouse α-Syn: hSyn and mSyn, respectively; SynF1 = fibrillar α-Syn; Syn1 = non-fibrillar α-Syn. For description of (**A**–**C**) see text. Scale bar: 10 µm.

Since the NIIs shown here were triggered by the addition of 1B fibrils, they could either correspond to seeded inclusions, or to a simple nuclear accumulation of the exogenous fibrils following their internalization and phosphorylation. We answered this question using different species-specific anti-α-Syn antibodies. Figure 5A shows a typical phospho-S129 α-Syn-positive “two-beam” NII (pSyn, red) at DIV30 in a neuron treated with human 1B fibrils at DIV7. The anti-human α-Syn antibody [69] (hSyn, green) shows that the NII does not coincide with the distribution of the residual 1B fibrils (green) that are absent in the untreated control (Ctrl. column) and that are scattered on the whole neuronal surface and in some poorly delineated perikarial regions (empty arrowheads, probably the late endolysosomal compartment [70]). This shows that the NIIs do not correspond to an accumulation of the input fibrils.

In agreement with this result, Figure 5B shows a “three-beam” NII and three neuritic inclusions revealed with a rodent-specific anti-α-Syn antibody [32] (mSyn, green) and the conformation-selective anti-α-Syn antibody [71] SynF1 (red). Figure 5B indicates that both the neuritic inclusions and the NII are made of endogenous α-Syn. The rodent-specific anti-α-Syn antibody (mSyn, green) that stains all the physiological pools of endogenous α-Syn (oligomers in synapses (empty arrowheads) and soluble monomers in cytoplasm and nucleoplasm) also intensely stains the neuritic inclusions and the NII. In addition, both the NII and the neuritic inclusions are strongly positive to SynF1 (red) while the physiological α-Syn pools are not. Since SynF1 has a high affinity for amyloid α-Syn assemblies over oligomeric and monomeric species [15,71], this shows that like the neuritic inclusions, the NIIs are amyloid inclusions.

Confirming this result, NIIs are invisible to the conformation-dependent antibody Syn1 [15,72] (Figure 5C). Indeed, oppositely to SynF1, Syn1 only detects physiological monomeric and oligomeric forms of α-Syn and not its amyloid assemblies [15,72]. Here, Syn1 only evidences the diffuse cytosolic/nucleoplasmic distribution of monomeric α-Syn and the pre-synaptic concentration of physiological α-Syn (empty arrowheads), while, as highlighted by the line scan in the overlay (LS, inset graph), the NII is Syn1-invisibile.

In conclusion, Figure 5 shows that NIIs are seeded inclusions made of endogenous α-Syn induced to self-assemble into intranuclear amyloid structures.

**Figure 6:**
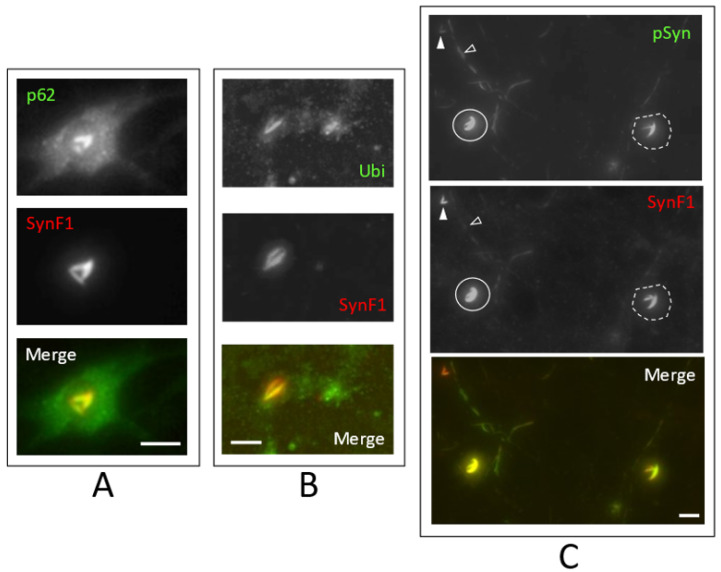
The 20× HCA images of 30 DIV mouse primary cortical neurons treated at DIV7 with 1B fibrils. SynF1: fibrillar α-Syn; Ubi: ubiquitin; pSyn: phospho-S129 α-Syn. For description of (**A**–**C**) see text. Scale bar: 10 µm.

NIIs induced by 1B are also positive to classical secondary markers of α-Syn aggregation that are routinely used to characterize pathological α-Syn inclusions [73,74,75]. Figure 6A shows a “three-beam” α-Syn NII (revealed with SynF1, red in overlay) that is also strongly positive for p62 (green in overlay). While this protein is classically found associated with the NCIs of α-Synucleinopathies and of various other aggregopathies [74], it is worth noting that in the nucleus, p62 condensates form a hub for proteasome-mediated protein catabolism [76]. Ubiquitination is also a classical modification found associated with NCIs in all α-Synucleinopathies [73,74,75]: Figure 6B shows a “two-beam” NII revealed with SynF1 which is also strongly positive for ubiquitin.

Comparison of the relative signals of SynF1 (red) and phospho-S129 α-Syn (green) in Figure 6C shows that the stoichiometry between the phosphorylation of S129 (pSyn) and the acquisition of a distinctive conformational epitope (SynF1) is not a constant in the α-Syn amyloids that are seeded by 1B and that populate the NIIs and the neuritic inclusions. Indeed, NIIs can be SynF1 “high” and pSyn “low” (plain arrowhead), SynF1 “high” and pSyn “high” (plain perimeter), or SynF1 “low” and pSyn “high” (dotted perimeter). One of the neuritic inclusion found in this field is pSyn positive but barely detected by SynF1 (empty arrowhead).

**Figure 7:**
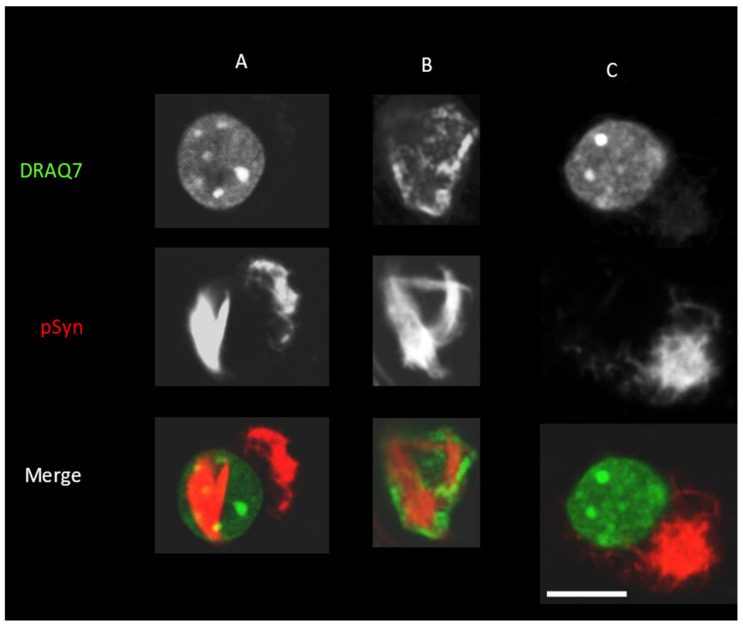
LSCM optical sections of 30 DIV mouse primary cortical neurons treated at DIV7 with 1B fibrils. Merge: DNA (DRAQ7) coded in green, phospho-S129 α-Syn (pSyn) coded in red. For description of (**A**–**C**) see text. Scale bar: 10 µm.

With regards to their size and their intranuclear localization, we presumed that NIIs could cause a chromatin reorganization. Figure 7 shows confocal sections of three neurons stained with the DNA-intercalating dye DRAQ7 and for phospho-S129 α-Syn. In Figure 7A, the neuron harbors both an NII and an NCI. Its nuclear chromatin morphology appears globally normal with, however, a slight DRAQ7 hyposignal that could correspond to the bed of certain regions of the NII. Figure 7B shows a neuron harboring a “three crescent moons” NII that not only pushes apart the chromatin but also heavily stretches the nucleus. Note that this type of nuclear deformation is also seen with Lamin staining at the tip of one of the beams constituting the NII cross of the video abstract (https://doi.org/10.5281/zenodo.6338439). However, no distinctive morphological signs of nuclear apoptosis are seen here in spite of the extensive chromatin remodeling (Figure 7B). For the sake of comparison, Figure 7C shows an NCI-only neuron with a morphologically normal nucleus. Though intriguing, the absence of apoptosis in all these neurons should have, however, been expected: seeded α-Syn inclusions are the result of a progressive stacking of α-Syn into amyloid assemblies. The α-Syn serving to “build” the inclusions is continuously “provided” by the host neuron, i.e., a process incompatible with cell death. Thus, to reach this size, the seeded inclusions must be accommodated by the host neuron for extended periods of time. In other words, the growth of α-Syn amyloids is probably conditional upon neuronal tolerance mechanisms.

**Figure 8:**
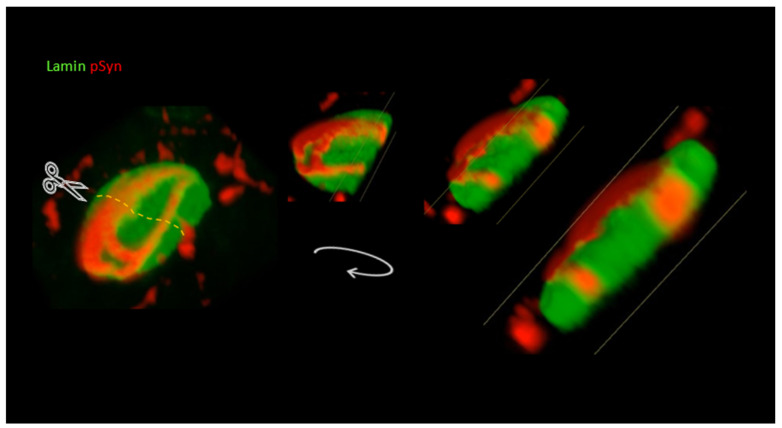
A 3D reconstruction, in silico sectioning (scissors/dots), and 3D rotation of a Z-stack of LSCM optical sections of a single 30 DIV mouse primary cortical neuron treated at DIV7 with 1B fibrils. Nuclear Lamin coded in green; phospho-S129 α-Syn (pSyn) coded in red showing an NII.

As for NIIs, this neuronal tolerance was apparently so high that α-Syn NIIs could even often outgrow the nuclear envelope—internally delineated by Lamin—to partially protrude in the extranuclear space. Figure 8 shows different angles of view of the 3D volume rendering of a Lamin (green) and phospho-S129 α-Syn (red) Z-stack of an NII. To appreciate the intranuclear/extranuclear transition of the inclusion, an in silico sectioning of the 3D volume was performed (dots, scissors). This reconstruction shows that while two extended portions of the NII beams remained essentially buried inside the nucleus (red), the convergence zone of the beams seemed to have “eroded” the nuclear Lamin envelope (green) and to have exposed its outer surface to the extranuclear space (black) (note the concomitant presence of several α-Syn NCI foci at distance from the nucleus).

We showed that α-Syn NIIs seeded by 1B can be made of one, two or three thick lentiform beams that are either strictly intranuclear or seem to outgrow the nucleus boundaries to eventually also protrude into the extranuclear space. We showed that NIIs can often coexist with perinuclear NCIs without apparent connections between the two inclusions.

**Figure 9:**
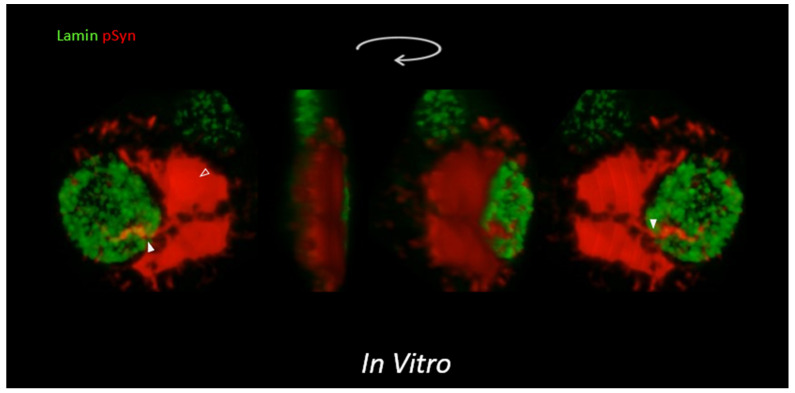
A 3D reconstruction and rotation of a Z-stack of LSCM optical sections of a single 30 DIV mouse primary cortical neuron treated at DIV7 with 1B fibrils. Nuclear Lamin coded in green; phospho-S129 α-Syn (pSyn) coded in red showing a bifid NCI (empty arrow) plus NII (plain arrow). Scale bar: 10 µm.

However, we also encountered a few neurons in which a main NCI was “sending” a thin aggregate process into the nuclear mass forming a filamentous NII. Figure 9 shows a neuron harboring a bifid α-Syn NCI revealed with phospho-S129 α-Syn (red, empty arrowhead) that extends an aggregate process (red, plain arrowhead) deep inside the nucleus (revealed by Lamin, green), thus forming a filamentous NII (plain arrowhead).

### 3.5. α-Syn NIIs Seeded In Vivo in Mice after Stereotaxic Injections of 1B Fibrils

This type of continuity between NCI and NIIs is precisely what we often observed in the brain of mice stereotaxically injected with 1B fibrils and which developed a widespread α-Syn pathology (see our previous comparative description and quantification of the α-Syn pathology induced by 1B fibrils in vivo vs. sham-injected animals [15] and Supplementary Figures S7 and S9).

**Figure 10:**
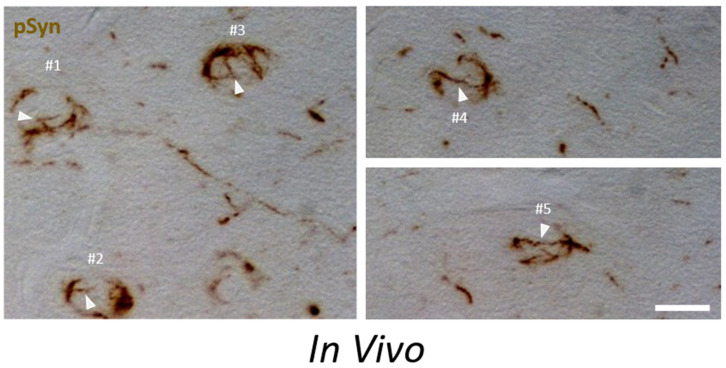
IHC of the α-Syn pathology in a mouse injected 1 month before with 1B fibrils. Paraffin section. Phospho-S129 α-Syn: brown. Zone injected with 1B: right striatum, zone sampled in the images: ipsilateral entorhinal cortex. Five neurons (#1 to #5) bearing NIIs (plain arrowheads) and NCIs are shown. For description see text. Scale bar: 10 µm.

Phospho-S129 α-Syn immunohistochemistry was performed on a thin semi-horizontal brain section (Supplementary Figure S7) without nuclear counterstain in order to avoid possible confounding of chromatic effects. Figure 10 shows a montage of 40× bright field images sampled at the level of the entorhinal cortex of an animal injected 1 month earlier with 1B fibrils in the medial part of its ipsilateral striatum.

Apart from the presence of numerous neuritic inclusions (note the axon extending from neuron #1 and containing many of these inclusions), the section plane intercepts five cortical neurons harboring typical phospho-S129 α-Syn-positive NCIs that wrap and delineate a significant portion of the outer perimeter of the sectioned nuclei. The presence of incomplete NIIs extending from the NCIs (such as in Figure 9) is noted for neurons #1, #2 and #3 (plain arrowheads). Neuron #3 shows three NII filaments converging on two small circular intranuclear structures. Neurons #2, #4 and #5 show a particular equatorial organization of the NCIs with two diametrically opposed “hotspots”. Interestingly, a partial NII extends from one of these hotspots in neuron #2, and a complete NII entirely bridges the two hotspots in neurons #4 and #5, forming “cat’s eye” images. Comparable images observed in the presence of a nuclear counterstain are shown in Supplementary Figure S8.

**Figure 11:**
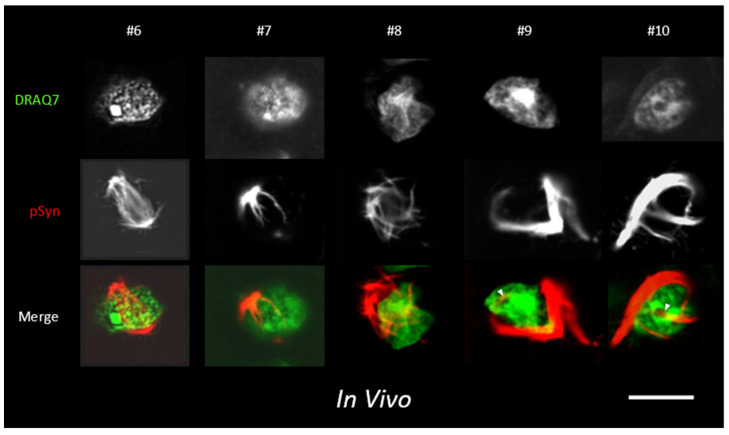
LSCM optical sections of the α-Syn pathology spread in a mouse injected 4 months before with 1B fibrils. Phospho-S129 α-Syn: coded in red; DNA/DRAQ7: coded in green. Zone injected with 1B and sampled by LSCM: right substantia nigra. Five individual nigral neurons (#6 to #10) bearing NCIs and NIIs are shown. For description see text. Scale bar: 10 µm.

Confocal imaging of thick brain cryosections (Supplementary Figure S9) reveals a similar type of organization between NCIs and NIIs in nigral neurons 4 months after an injection of 1B fibrils above the ipsilateral substantia nigra pars compacta [Figure 11, Supplementary video S1 (https://doi.org/10.5281/zenodo.6338718)]. DRAQ7 was used to stain the nuclear chromatin. Neurons #6 and #7 show the typical pattern of inclusion as found in the entorhinal neurons #2, #4 and #5 in Figure 10. Diametrically opposed α-Syn NCI hotspots bridged intranuclearly by a filamentous NII. Neuron #8 shows an NCI–NII continuum taking the form of a spinous corona that severely deforms the nucleus. Neurons #9 and #10 can be compared to the entorhinal neuron #3 in Figure 10, in which NCIs extend NII projections that intercept/stab circular intranuclear structures characterized by a hyposignal with DRAQ7 (plain arrowheads, Figure 11). Supplementary video S1 (https://doi.org/10.5281/zenodo.6338718) shows another crosswise NII that diametrically bridges two regions of an NCI partially enveloping the nucleus of a nigral neuron. It is clearly visible that the NII bridge pushes apart the chromatin, creating a DNA-free tunnel (low DRAQ7 signal) along its diametral course across the nucleus. Note the presence of a healthy bystander neuron without inclusion.

This illustrates how 1B fibrils injected in vivo are particularly prone to seed an α-Syn pathology in which both NCI and NII are coexisting in the same diseased neuron, the two inclusion types forming a continuum with many α-Syn inclusion processes extending across the nuclear boundary and bridging NIIs and NCIs. This difference with the in vitro situation in which many “pure” NIIs are observed after seeding with 1B might be due to the fact that in vitro, every neuronal soma is directly exposed to 1B seeds, increasing the probability that exogenous fibrils quickly reach the nucleus after uptake and seed there the buildup of a “pure” NII. In vivo, the synucleinopathic neurons observed are distant from the 1B fibril injection site (especially the entorhinal cortex neurons in Figure 10), and the inclusions observed in their somas derive from the intraneuronal retrograde spread of an aggregative process initiated distally in their pre-synaptic terminals [77]. In this case, it seems likely that NCIs are formed first, with α-Syn fibril fragments secondarily leaking out from the NCI and reaching the intranuclear space to seed NIIs.

**Figure 12:**
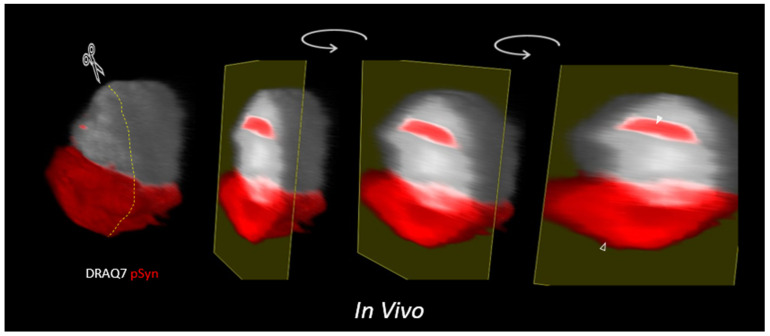
A 3D reconstruction, in silico sectioning (scissors/dots), and 3D rotation of a Z-stack of LSCM optical sections of a single nigral neuron bearing an NII (plain arrow) and an NCI (empty arrow) in a mouse injected 4 months before with 1B fibrils. Phospho-S129 α-Syn: coded in red; DNA/DRAQ7: coded in white. Zone injected with 1B and sampled by LSCM: right substantia nigra.

It is for the moment difficult to tell how the continuity between NCIs and NIIs becomes progressively established. Are NIIs formed as nuclear extensions of NCIs or do NIIs and NCI build up independently and fuse at some point? Figure 12 and the corresponding Supplementary video S2 (https://doi.org/10.5281/zenodo.6338733) show a nigral neuron harboring both an NCI and an NII beam without any apparent connections: this would tend to favor the second hypothesis.

## 4. Discussion

Our in vitro and in vivo results characterize the first known synthetic human α-Syn fibril strain endowed with the ability to seed NIIs [15]. This property has not been reported for any of the synthetic α-Syn fibrils characterized so far [8,26,28,32,36,62,63,67,68] and the amyloid fold of this new strain can be distinguished from previous synthetic α-Syn fibrils (Supplementary Figure S1). This unique capability of 1B to seed NIIs echoes the fact that the only human α-Synucleinopathy in which α-Syn NIIs are observed is MSA [50]. The parallel is further reinforced by the findings that the 1B strain and the fibrils amplified from MSA patients share a very poor detectability by ThT compared to regular “Type 2” fibrils (Supplementary Figure S6) [15] or to fibrils amplified from PD patients [51].

This body of evidence supports the hypothesis that the 1B strain shares unique architectural and/or functional characteristics with the MSA α-Syn fibrils [27,34]. From a mechanistic point of view, our data also indicate that the seeding of α-Syn NIIs does not need any upstream alterations of “third party” neuronal mechanisms. This capability is therefore integrally encoded in the structure of 1B. It is thus tempting to speculate that the α-Syn fibrils underlying MSA behave similarly.

Importantly, the only other amyloid aggregopathy presenting lentiform NIIs with a “cat’s eye” marble appearance is a subset of frontotemporal dementias (FTD) [50] involving TDP-43 [78]. This latter disease subset involves the fibrillogenic protein TDP-43 [79], but also concomitant mutations of either the Progranulin gene (Type A) or of the VCP gene (Type D) [78]. Without the latter mutations, lentiform TDP-43-positive NIIs are not observed [50]. This indicates that at variance from the synthetic α-Syn fibril strain 1B which is autonomous in that respect, TDP-43 fibrils in FTD cannot seed lentiform NIIs on their own. Thus, it seems likely that contrary to FTD and TDP-43, the early and pathognomonic formation of α-Syn NIIs in MSA [43,44,45,48] is entirely α-Syn-fibril-strain-dependent.

It is interesting to note that in MSA, the formation of NIIs is an early event preceding the apparition of glial cytoplasmic inclusions (GCIs) in oligodendrocytes [43,44,45]. At 1- and 4-months post-injection we did not spot the presence of GCIs in mice but performing in vivo experiments with extended observation durations should allow to detect the delayed formation of experimental GCIs [47]. Work is also in progress to further decipher the atomic organization of the 1B fibrils and to identify the cascade of molecular events prevailing to the intranuclear seeding of NIIs. Due to their small size (14 kDa), α-Syn monomers and small oligomers (<4 mer) can passively diffuse through the nuclear pore sieve and gain access the nucleoplasm [80,81]. However, regarding amyloid fibril seeds, specific transport mechanisms relying on karyopherins [82,83] are in all likelihood involved. Once in the nucleus, possible interaction of the seeds with PML bodies [84] or histones [80], and facilitation of NII growth by nuclear liquid–liquid phase separation phenomena [85], are research directions that will help understand why only specific α-Syn amyloid strains can colonize the nuclear compartment.

## Data Availability

The data presented in this study are available on request from the corresponding authors.

## Supplementary Materials

The following supporting information can be downloaded at: https://www.mdpi.com/article/10.3390/biom12030436/s1, Figures S1–S9; Supplementary video S1 (also available at: https://doi.org/10.5281/zenodo.6338718): Crosswise α-Syn NII that diametrically bridges two regions of a NCI partially enveloping the nucleus of a mouse nigral neuron 4 months after in vivo injection of 1B fibrils; Supplementary video S2 (also available at: https://doi.org/10.5281/zenodo.6338733): 3D volume reconstruction a mouse nigral neuron harboring both a NCI and a single beam NII without any apparent connections, 4 months after in vivo injection of 1B fibrils; Supplementary video S3 (also available at: https://doi.org/10.5281/zenodo.6338439): Video Abstract.
