# Supplementary material for: Neurons with Cat’s Eyes: A Synthetic Strain of α-Synuclein Fibrils Seeding Neuronal Intranuclear Inclusions
Source: Biomolecules. 2022 Mar 11;12(3):436. doi: 10.3390/biom12030436 (PMC8946814; doi:10.3390/biom12030436)
Supplement: Supplementary file 1 [file biomolecules-12-00436-s001.zip › biomolecules-1610392-supplementary/biomolecules-1610392 Supplementary Figures S1,S2,S3,S4,S5,S6,S7,S8,S9.pdf]

Supplementary FIGURES

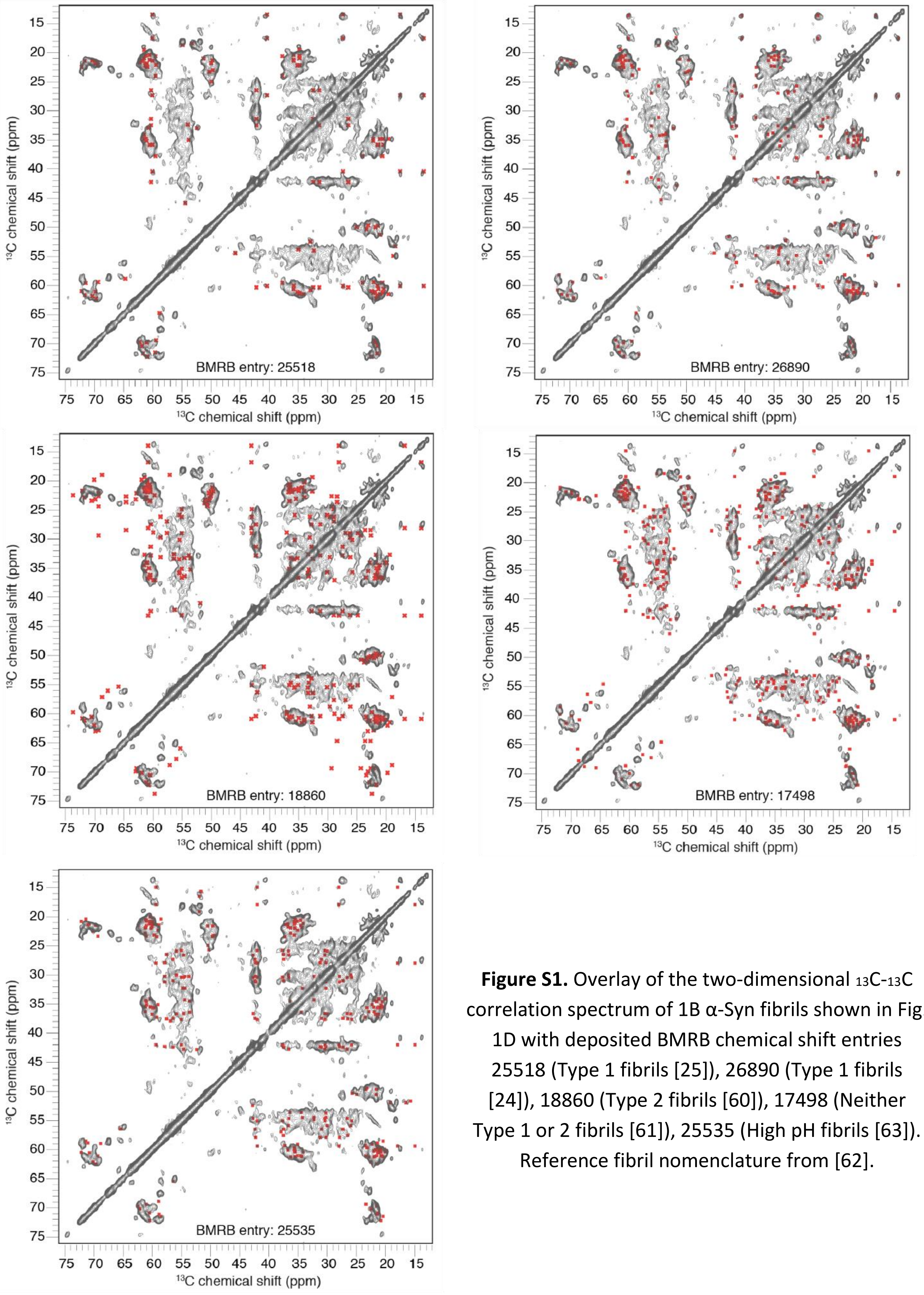

Figure S1. Overlay of the two-dimensional ^{13}C-^{13}C correlation spectrum of 1B α-Syn fibrils shown in Fig. 1D with deposited BMRB chemical shift entries 25518 (Type 1 fibrils [25]), 26890 (Type 1 fibrils [24]), 18860 (Type 2 fibrils [60]), 17498 (Neither Type 1 or 2 fibrils [61]), 25535 (High pH fibrils [63]). Reference fibril nomenclature from [62].

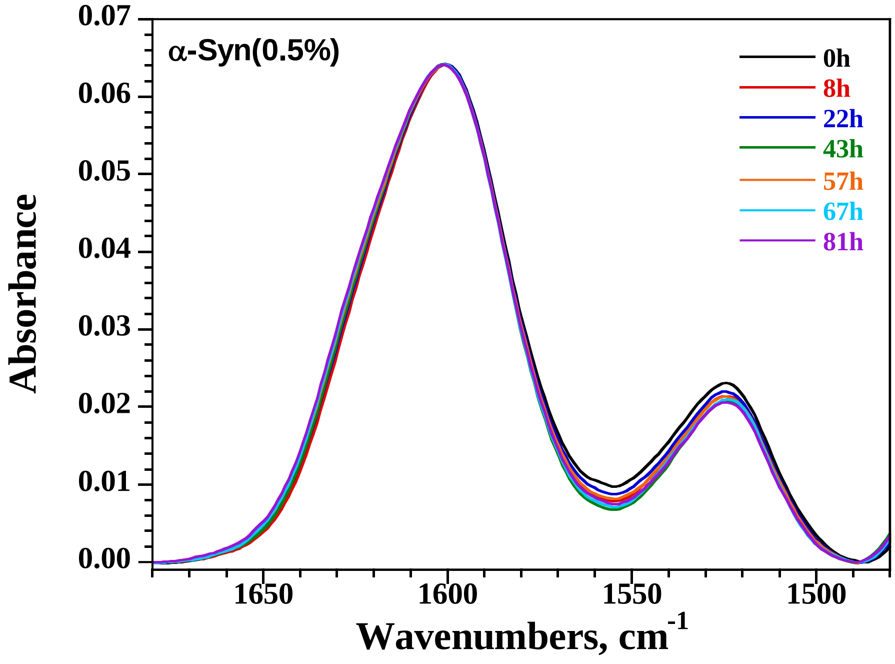

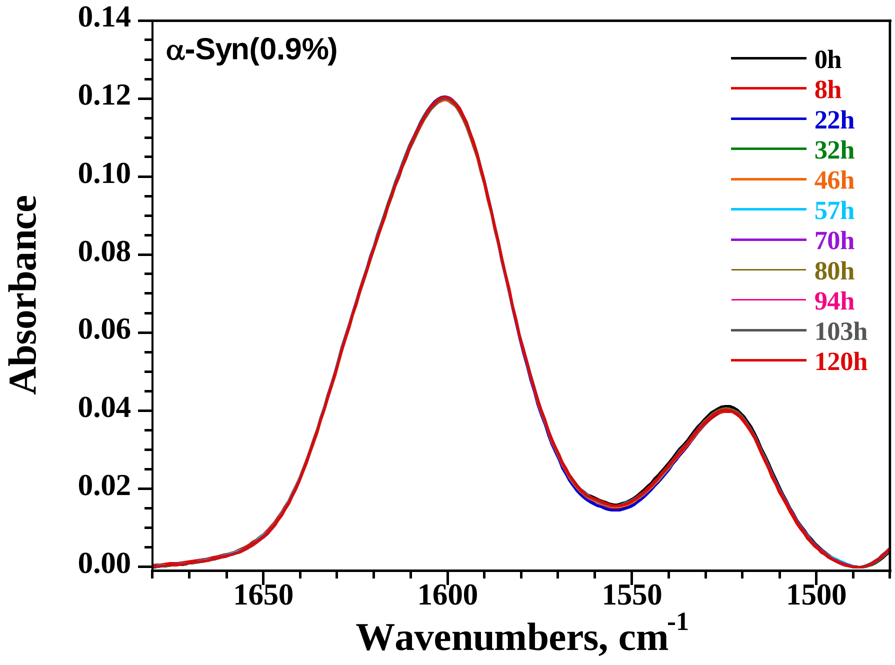

Figure S2. IR spectra in the amide I' and amide II' region of monomeric α-Syn expressed with 13C at a concentration of 5 mg/ml (top) and 9 mg/ml (bottom).

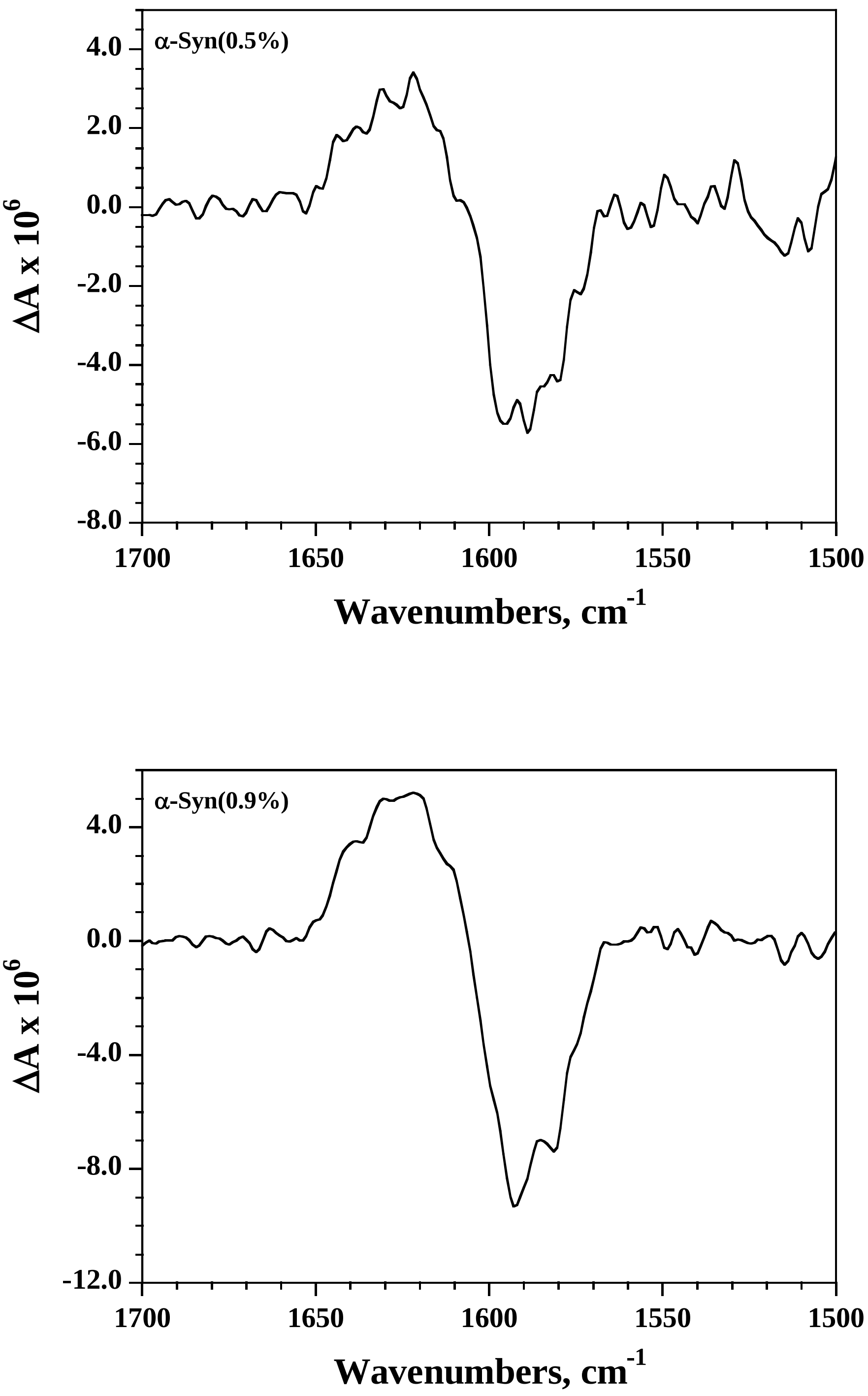

Figure S3. VCD spectra of monomeric α-Syn expressed with 13C at a concentration of 5 mg/ml (top) and 9 mg/ml (bottom). ΔA represents the difference of IR absorbances under left and right circularly polarized light.

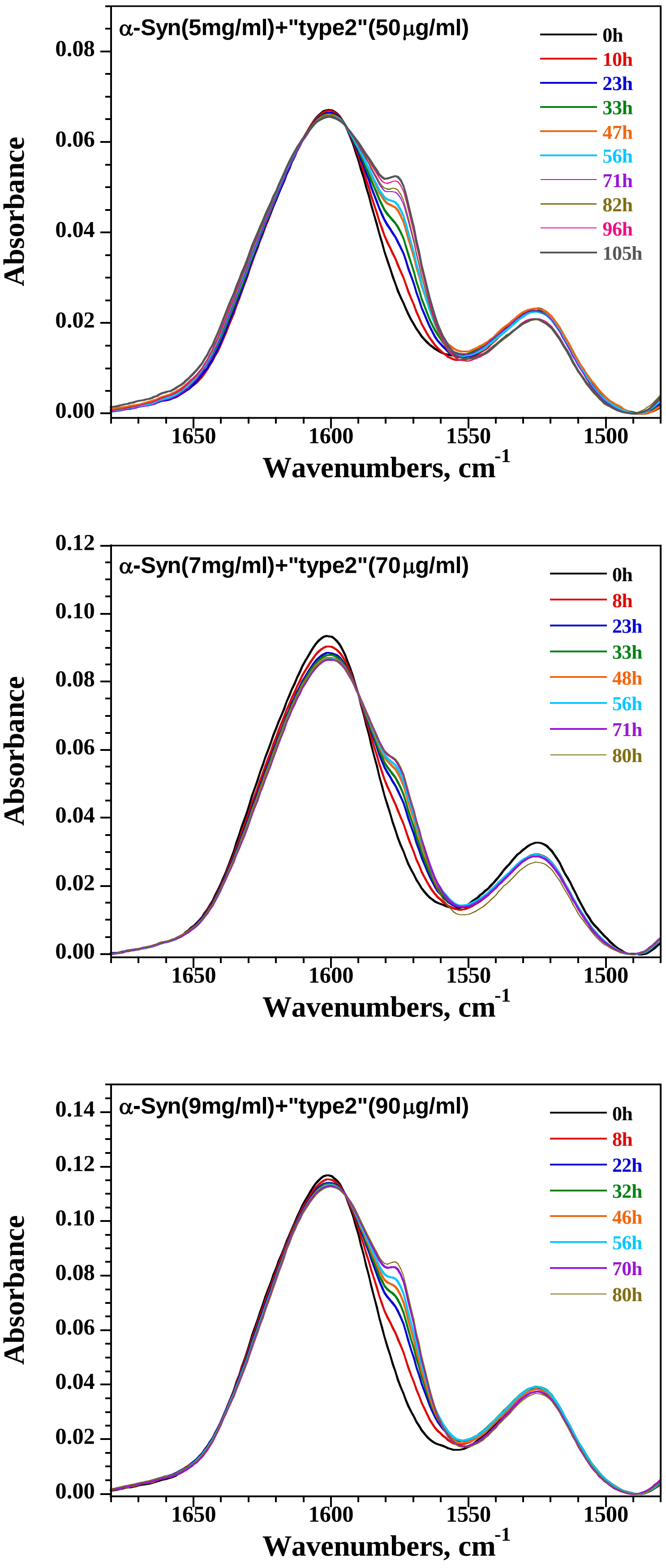

Figure S4. IR spectra of monomeric α-Syn at a concentration of 5 mg/ml (top), 7 mg/ml (medium) and 9 mg/ml (bottom) seeded with 1% of the common "type 2" human α-Syn fibril strain.

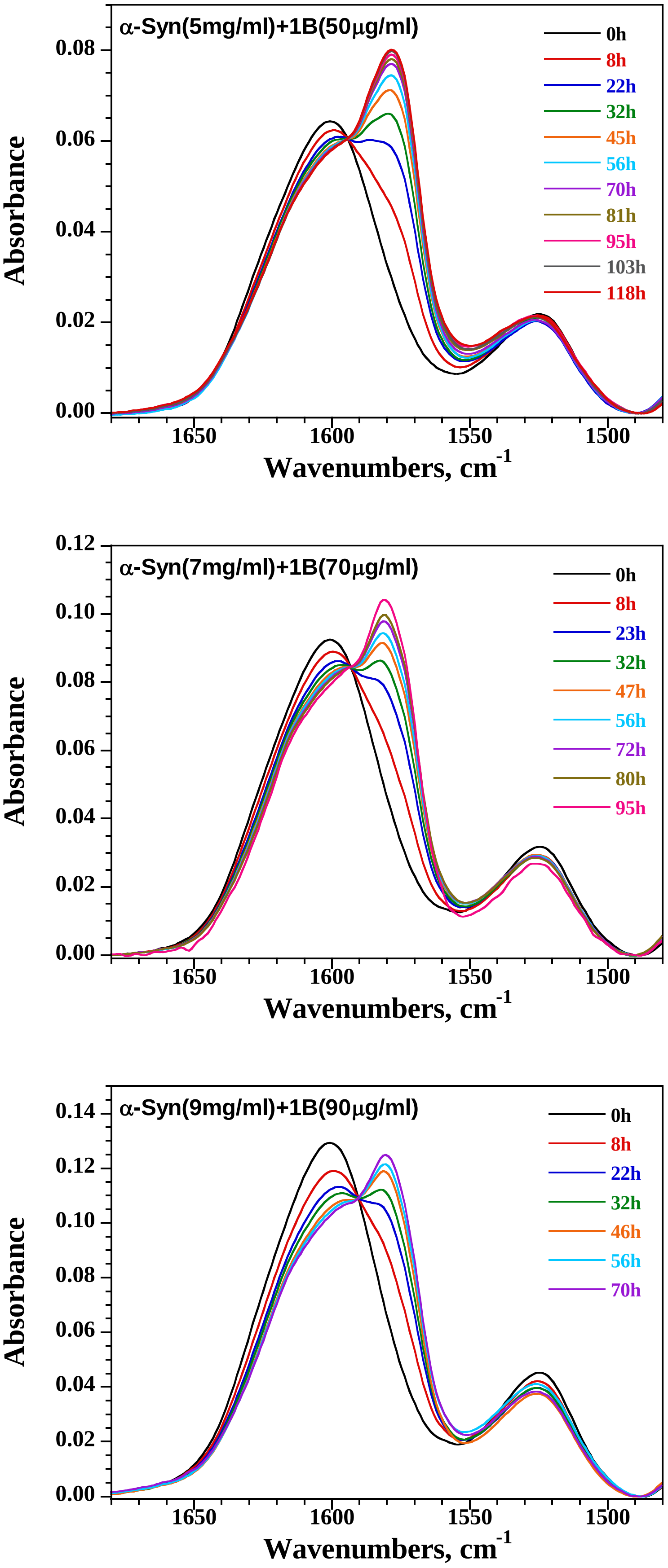

Figure S5. IR spectra of monomeric α-Syn at a concentration of 5 mg/ml (top), 7 mg/ml (medium) and 9 mg/ml (bottom) seeded with 1% of the 1B α-Syn fibril strain.

ThT

Saline	476	486	488
α-Syn mono	490	502	499
Type2 α-Syn fibrils	25075	25125	25101
1B α-Syn fibrils	2432	2458	2420

SG

Saline	1432	1406	1429
α-Syn mono	1860	1855	1823
Type2 α-Syn fibrils	6523	6441	6510
1B α-Syn fibrils	32508	35011	34120

X-34

Saline	4333	4520	4478
α-Syn mono	5995	5780	6045
Type2 α-Syn fibrils	45020	46899	45798
1B α-Syn fibrils	43960	46664	42760

Figure S6. Comparative analysis of Type 2 fibrils, 1B α-Syn fibrils, α-Syn monomers, and solvent (saline) using 3 fluorescent amyloid probes: Thioflavin T (ThT), SybrGreen 1 (SG) and X-34. For each sample, 3 measurement replicates performed with every single probe are shown. Data in arbitrary fluorescence units. Data cell color depth linearly follows the numerical values, the higher the darker. Detections on 0.1 mg/ml α-Syn for all samples except saline (no α-Syn). Probe concentrations: ThT: 20 µM, X-34: 20 µM, SG: 1/1000 commercial stock. Full description of methods in [15]. While X-34 does not discriminate between Type 2 and 1B fibrils and indicates the similar amyloid contents of the two fibril samples, ThT shows a 10 times higher signal for Type 2 fibrils compared to 1B. At the opposite, SG shows a 5 times higher signal for 1B fibrils compared to Type 2. This indicates that the two fibril types offer different solvent-accessible surface topologies and differentially bind the two probes, see also [15].

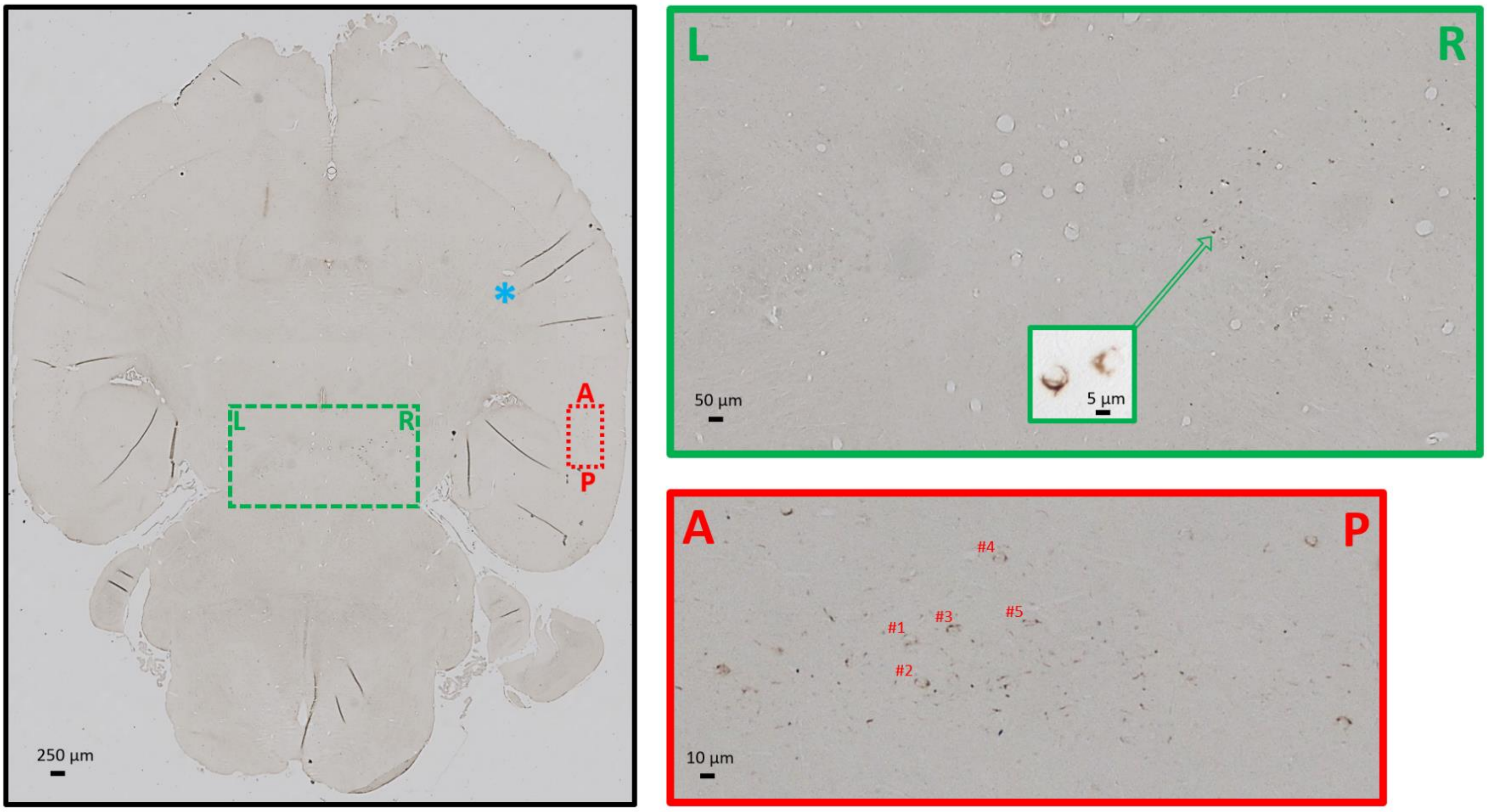

Figure S7. Bright-field images of a semi-horizontal paraffin section (10 µm-thick) of the brain of a mouse injected 1 month earlier with 1B α-Syn fibrils in the right dorsal striatum. The dorso-ventral tilt angle of the section plane allows to intercept the substantia nigra (SN) posteriorly (green region, L: left, R: right) as well as the striatum anteriorly (blue asterisk: ventral projection of the injection site). Phospho-S129 α-Syn immunohistochemistry without counterstain. The red box is sampling the right entorhinal cortex (A: anterior, P: posterior). The zoomed SN regions (green box) shows that the striatal injection triggered the retrograde formation of inclusions in several nigral neurons (see the focused zoom inset) located in the ipsilateral SN (VTA). No inclusions are seen in the contralateral SN. Many inclusions (Neuritic, NCIs, NIIs) are seen in the entorhinal cortex (red box). The neurons numbered from #1 to #5 are shown in main Figure 10.

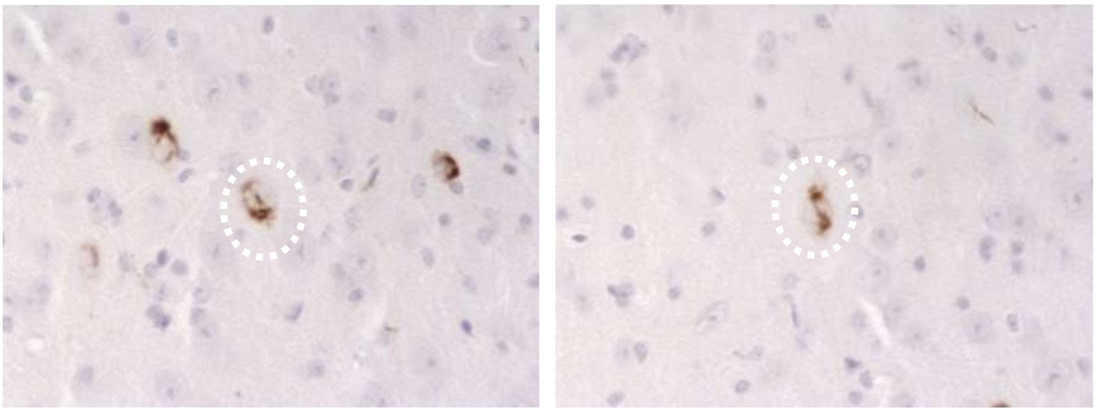

Figure S8: 40x bright field images sampled at the level of the VTA of an animal injected 1 month earlier with 1B fibrils in the medial part of its ipsilateral striatum. Paraffin section. Phospho-S129 α-Syn immunohistochemistry + Haematoxilin-Eosin counterstain. The circled neurons show "Cat's Eye" NIIs connecting diametrally opposed NCI hotspots.

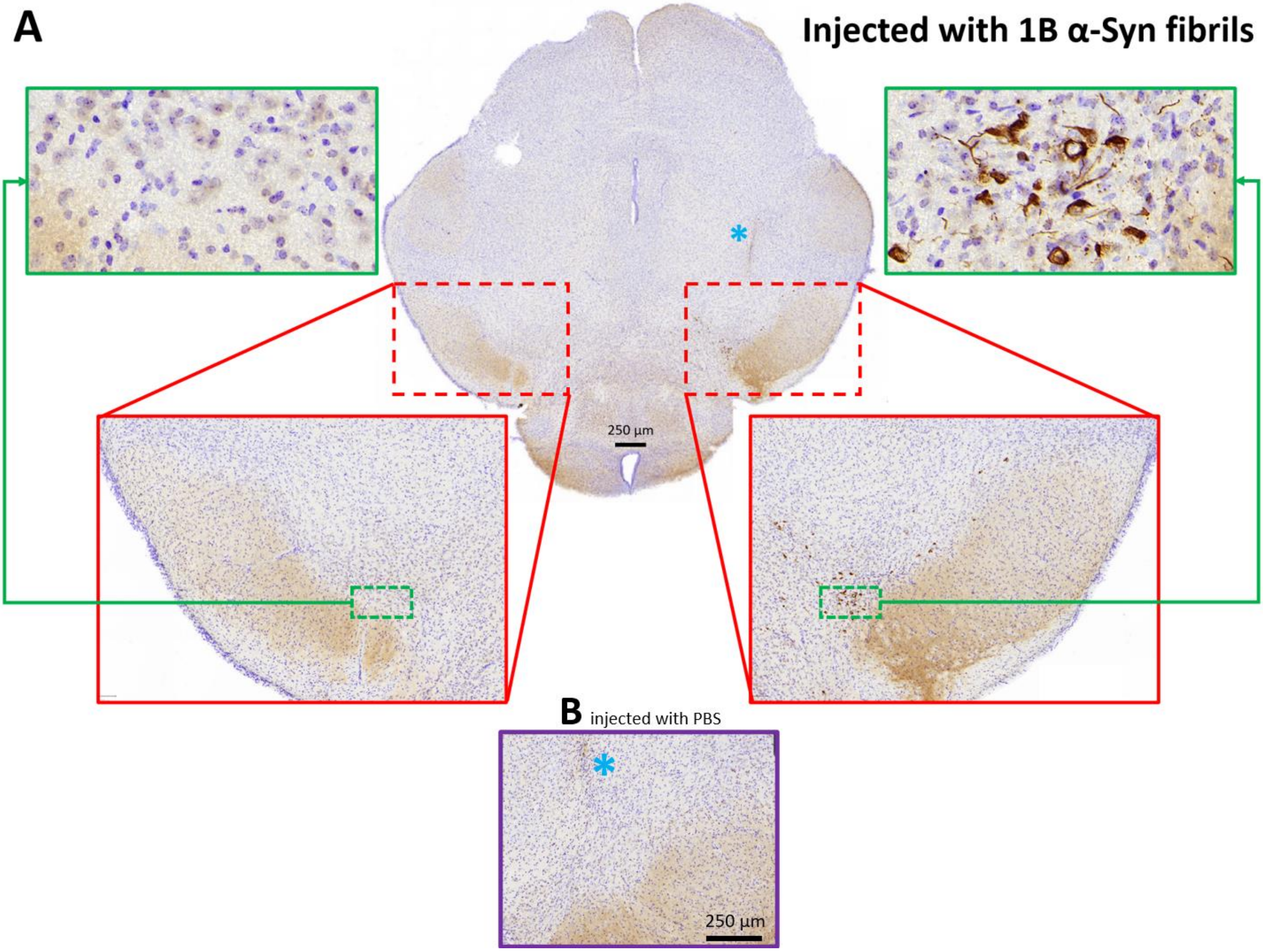

Figure S9. Bright-field images of coronal cryosections (50 µm-thick) of the midbrain of mice injected 4 months earlier with 1B fibrils above the right substantia nigra (SN) **(A)** or with solvent alone (PBS) **(B)**. Phospho-S129 α-Syn immunohistochemistry with cresyl violet counterstain. **(A)** The red boxes are sampling the right and left SNs easily recognizable due to the presence of background IHC stain in the pars reticulata. The blue asterisk indicates the midbrain scar caused by the injection needle along its course. Low and intermediate magnifications (red boxes) as well as zoomed sub-regions (green boxes) evidence many nigral neurons bearing α-Syn inclusions on the side of the injection. Controlateral SN or other regions of the midbrain section show no inclusions. Due to the thickness of the section, neuritic inclusions are very easily recognized, but at the same time, sorting out the many somatic inclusions as NCIs or NIIs is hardly possible. For this reason, immunofluorescence and laser scanning confocal microscopy was used to optically subsection this type of sample (main article Figures 11 & 12, and supplementary movies 1 & 2). **(B)** Purple box: close-up of the right SN in a PBS-injected animal. Blue asterisk: injection scar. No inclusions are seen above the background IHC stain (mostly prominent in the pars reticulata, bottom right corner). In these experimental conditions, sham-injected animals never develop α-Syn pathology [15].
