# Supplementary material for: Neurons with Cat’s Eyes: A Synthetic Strain of α-Synuclein Fibrils Seeding Neuronal Intranuclear Inclusions
Source: Biomolecules. 2022 Mar 11;12(3):436. doi: 10.3390/biom12030436 (PMC8946814; doi:10.3390/biom12030436)
Supplement: Supplementary file 1 [file biomolecules-12-00436-s001.zip › biomolecules-1610392-supplementary/biomolecules_1610392 VideoLegends.pdf]

Legends to videos files for biomolecules-1610392

- Legend to <u>Video Abstract :</u>
- **23-day-old α-Syn Neuronal Intranuclear Inclusion (NII) seeded by the synthetic α-Syn amyloid strain 1B in a cultured mouse cortical neuron.** Animation of a 3D volume rendering of a nuclear Lamin (white)/phospho-S129 α-Syn (red) Z-stack. A "2-beam" NII forming a cross spans the whole nuclear volume. In this movie the Lamin channel is periodically switched on & off to let the observer appreciate the 3D organization of the NII embedded in the nucleus. Max diameter of the nucleus (Lamin, white) is 11 μM.
-
- Legend to <u>Supplementary video S1 :</u>
- **Crosswise α-Syn NII that diametrically bridges two regions of a NCI partially enveloping the nucleus of a mouse nigral neuron 4 months after *in vivo* injection of 1B fibrils**. The movie shows a full 360° animated series of the 3D volume of a nigral neuron soma marked for phospho-S129 α-Syn (red) and DNA using DRAQ7 (white). The neuronal soma was chopped in silico to reveal a section plane going through the crosswise portion of the NII. The series is shown for the overlay volume (white and red), for the α-Syn inclusions volume only (red), and for the DNA volume only (white). It is clearly visible that the NII bridge pushes apart the chromatin, creating a DNA-free tunnel (low DRAQ7 signal, white) along its diametral course (nuclear dimeter at the level of the in silico section: 9μm) . Note the presence of a healthy bystander neuron without inclusion.

- Legend to <u>Supplementary video S2:</u>
- **3D volume reconstruction a mouse nigral neuron harboring both a NCI and a single beam NII without any apparent connections, 4 months after *in vivo* injection of 1B fibrils**. DNA is marked with DRAQ7 (white) and phospho-S129 α-Syn is in red. The 3D volume was animated (360° rotation), and the nuclear DNA signal was cyclically switched on & off to let the observer appreciate the NII and NCI 3D morphologies. Nucleus max. diameter (DRAQ7, white): 13μM.
